# Differentiating right upper limb movements of esports players who play different game genres

**DOI:** 10.1038/s41598-025-90949-6

**Published:** 2025-02-22

**Authors:** Antoine Dupuy, Mark J. Campbell, Adam J. Toth

**Affiliations:** 1 Lero the Research Ireland Centre for Software, University of Limerick, Limerick, Ireland; 2 Department of Physical Education & Sport Sciences, University of Limerick, Limerick, Ireland; 3 Health Research Institute, University of Limerick, Limerick, Ireland; 4 Centre for Sport Leadership, Stellenbosch University, Stellenbosch, South Africa

**Keywords:** Biomechanics, Kinematics, Performance, Physical demand, Video gaming, Motor control, Human behaviour

## Abstract

Esports is a fast-growing worldwide phenomenon encompassing hundreds of millions of competitive players. It is well-established that different game genres require distinct cognitive skills, but the biomechanical implications of playing different game genres have received little attention. This is the first study to quantify gamers’ kinematic behaviour across genres, demonstrating the importance of physical demands on performance and equipment in esports. The purpose of this study was to investigate whether the right upper limb kinematic behaviour differs among players across game genres. 63 esports players played a First Person Shooter (FPS), Multiplayer Online Battle Arena (MOBA), or Adventure game for 10 min. Three tri-axial accelerometers, positioned on each participant’s right upper limb (hand, forearm, arm), recorded kinematic data during gameplay. Hand acceleration magnitude, direction change, distance travelled (sum of all hand displacements over 10-min of gameplay), and displacement area (size and shape) were calculated in addition to hand, forearm, and arm acceleration ratios. There was a marked difference in movement patterns across players of different game genres. FPS players displayed greater hand acceleration magnitude (0.96 m.s^−2^ ± 0.07 SEM), moved their hand through a greater distance (38.96 m ± 2.47 SEM), and over a larger displacement area (119.13 cm^2^ ± 16.05 SEM) compared to MOBA and Adventure players. MOBA players exhibited greater hand acceleration magnitude (0.73 m.s^−2^ ± 0.05 SEM), changed direction more (2335 ± 172 SEM) and covered more distance (29.25 m ± 1.80 SEM) compared to Adventure players within a smaller overall area (70.49 cm^2^ ± 9.91 SEM). These findings have the potential to impact the design of gaming equipment and the training volumes of gamers across different game genres, so as to mitigate injury risk and improve overall gaming performance.

## Introduction

Esports are organized video game competitions that have been gaining research attention over the past few decades^1–3^. It is widely accepted that esports performance involves cognitive abilities that include attention, task-switching, information processing, and memory abilities to produce skilled motor outcomes^4–7^. There are over a thousand different competitive games^8^ currently, but no consensus about esports genre classification has been found yet^9,10^. However, it has been noted that the cognitive demands of esports vary between different genres^4,10–12^. For example, Campbell and colleagues highlight that visuo-motor coordination demands are high in First Person Shooter (FPS) games, compared to the greater value on strategic knowledge in Multiplayer Online Battle Arena (MOBA) games^4^. Despite the growing body of knowledge pertaining to the cognitive demands of esports, only recently has research been directed to better understanding the physical demands of gaming.

While our understanding about the movement mechanics of video game players is still in its infancy^13^, some initial studies exist demonstrating video gamers display elite bimanual coordination^14–16^, response times^4,7,17^ and dexterity^5,18^ when using their gaming peripherals (mouse and keyboard, controller, mobile phone, or arcade stick) to interact with the game^18,19^. Unlike exergaming and motion-based gaming (using full body motion to move in the virtual environment), PC video games primarily require multi-joint coordination and motor abilities of the upper limb^18,20–22^. Two recent studies highlight the importance of upper limb movements for gaming performance. Both Park et al., (2021) and Li et al., (2021), show that esports players with greater expertise have higher mean, median, and peak hand accelerations compared to lower skilled players^23,24^. While FPS games appear to place significant demands on upper limb motor control, no work has investigated how physical demands of video games differ across game genres, even though different game genres involve distinct actions and motions during gameplay^4,18,25–27^.

These distinctions in actions and motions may be tied to the gameplay (game scenario, environment, and rules) dictated by each game. Interestingly, FPS, MOBA and Adventure games are among the most popular genres in the gaming community, despite being inherently different in their gameplay. FPS games (e.g., Counter Strike 2, Valorant, Overwatch, Rainbow 6: siege) are among the most well-known video games worldwide^28,29^. FPS players navigate a 3-dimensional virtual environment through a first-person view and utilize different weapons to eliminate virtual opponents. Players need to control where they look, aim, and move through keyboard inputs and precise computer mouse movements^21,30^. MOBA games (e.g., League of Legends, Defense of the Ancients 2), on the other hand, are played in a virtual world viewed from a top-down perspective (isometric view) with a restricted field of view around the avatar ^31^. Due to this characteristic, MOBA players navigate their environment differently from FPS games since players must click on the screen location where they desire to move. MOBA games are thought to place a high demand on tactics and strategies along with a high number of actions per minute (APM)^31^. Finally, Adventure games (e.g., Minecraft, Assassin’s Creed, The Witcher III), which can be played from a first- or third-person perspective, are ‘open world’, meaning that players are free to explore vast virtual environments. The objective of adventure games is to explore the world and survive dangerous situations (monster encounters, manage basic character’s needs, etc.), which requires high levels of awareness and vigilance of the surroundings. Additionally, these games require superior task-switching abilities to manage stressful and unexpected situations. While the abovementioned functional differences among game genres are well documented, little to no attention has been allocated to addressing the biomechanical differences required to play these popular game genres. Addressing this gap will have important implications regarding the injury risk, sensory-motor training methods, and equipment optimisation for players of specific game genres^11^.

The purpose of this study is to determine whether players of different game genres display distinct upper limb kinematic behaviour. In this respect, we aimed to evaluate (i) how rapidly players move their hands by calculating the root mean square (RMS) of global hand acceleration, (ii) how players move their upper limb segments relative to one another by calculating the RMS acceleration ratios between the different limb segments (hand:forearm and forearm:arm RMS acceleration ratios), (iii) how often they change the direction of their hand movement, (iv) the cumulative distance through which their hand travels, and finally (v) over what surface area they move their hand over the course of 10-min of gameplay. We hypothesize that MOBA players exhibit quicker hand movements than FPS players, who in turn move quicker than Adventure players, as indicated by greater RMS hand acceleration. FPS players are presumed to engage their hand and forearm more, displaying higher hand-to-forearm and forearm-to-arm acceleration ratios than MOBA and Adventure players. MOBA players likely change hand movement directions most frequently, followed by FPS and Adventure players, based on a higher number of velocity zero crossings. MOBA players are also expected to travel greater hand distances than FPS and Adventure players, with FPS players travelling more distance than Adventure players. Lastly, FPS players’ hand movements are predicted to span larger mousepad areas than MOBA players, who cover more area than Adventure players, as shown by larger 95% confidence ellipses of hand displacement data.

## Results

The statistical results of all variables have been summarized in Table 1.

**Table 1:** Tests-statistic, p-values, and effects sizes of all variables of interest.

Variables	Comparisons	Test-statistic			P-value		Effect size
		**F**	**t**	**χ**^**2**^			**η**^**2**^
Root mean square (RMS) of global hand acceleration	A x B	**17.787**			≤ 0.001***	0.024*	**0.372**
	A x C					≤ 0.001***	
	B x C					0.002**	
Hand: forearm RMS acceleration ratios	A x B	**8.058**			≤ 0.001***	0.043*	**0.212**
	A x C					≤ 0.001***	
	B x C					0.102	
Forearm: arm RMS acceleration ratios	A x B	**4.414**			0.016*	0.299	**0.128**
	A x C					0.004**	
	B x C					0.096	
Hand global velocity signal zero-crossings	A x B	**21.468**			≤ 0.001***	0.376	**0.417**
	A x C					≤ 0.001***	
	B x C					≤ 0.001***	
Hand cumulative distance travelled	A x B	**13.873**			≤ 0.001***	0.01**	**0.316**
	A x C					≤ 0.001***	
	B x C					0.03*	
Hand displacement areas	A x B	**3.338**			0.042*	0.018*	**0.100**
	A x C					0.838	
	B x C					0.04*	
Long: Short displacement ratios	A × 1		**5.154**			≤ 0.001***	**0.172**
	B × 1		**3.073**			0.067	0.057
	C × 1		**1.977**			0.008**	**0.135**
DPI choice (400 DPI)	A x B			**11.626**	0.02*	0.05	0.029
	A x C					0.101	0.023
	B x C					0.529	0.004
DPI choice (1000 DPI)	A x B					0.052	0.029
	A x C					0.126	0.020
	B x C					0.472	0.004
DPI choice (1600 DPI)	A x B					0.002*	**0.078**
	A x C					0.01	0.053
	B x C					0.27	0.010

Comparisons code: A = FPS; B = MOBA; C = Adventure. One sample t-tests (A × 1; B × 1; C × 1) have been performed on the “Ratio of ellipse long and short axes” which represents the long and short axis of the 95% ellipse of confidence. *, ** and *** indicate significant differences at p ≤ 0.05, p ≤ 0.01 and p ≤ 0.001, respectively. Sidak’s correction has been applied to the contingency analysis (Chi-square) performed on the DPI choice proportion with a minimum significant level of p ≤ 0.006. η^2^ = 0.01 indicates a small effect; η^2^ = 0.06 indicates a medium effect; η^2^ = 0.14 indicates a large effect. Significant p-values, large effect sizes, and F-values, t-values, and χ^2^-values are displayed in bold.

### DPI choice

The DPI choice proportion was significantly different from an equal distribution between groups (X^2^ (4, N = 63) = 11.626, p = 0.02). FPS players were less likely to choose 1600 DPI compared to MOBA players (FPS = 8 (26.7%); MOBA = 12 (75%); p ≤ 0.002, η2 = 0.078). No significant difference was found between the distribution of 400 and 1000 DPIs across players from different game genres. Significant p-values, large effect sizes, along with F-values, t-values, and χ^2^-values are displayed in bold in Table 1.

### Hand acceleration

**Fig. 1:**
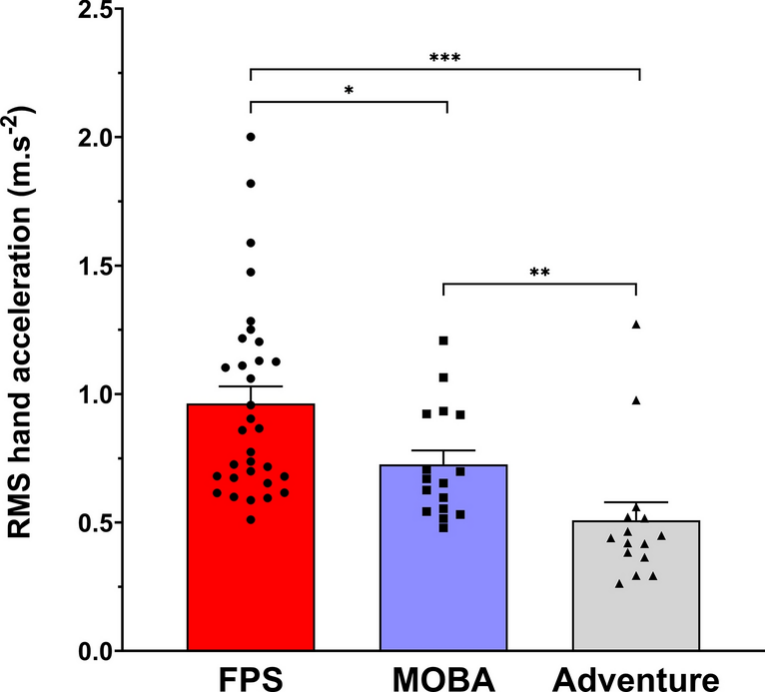
Root mean square (RMS) of global hand acceleration during the 10-min gameplay (mean ± SEM) for *FPS* (red), *MOBA* (blue), and *Adventure* (grey) players. *, ** and *** indicate significant differences at p ≤ 0.05, p ≤ 0.01 and p ≤ 0.001, respectively.

When examining the RMS of global hand acceleration, a main effect of game genre (F_(2,60)_ = 17.787, p ≤ 0.001, η^2^ = 0.372) was found (Fig. 1). Post hoc analysis revealed that FPS players had greater hand acceleration magnitude compared to MOBA (p = 0.024) and Adventure players (p ≤ 0.001). In addition, MOBA players had greater hand acceleration magnitude compared to Adventure players (p = 0.002).

### Upper limb acceleration ratios

**Fig. 2:**
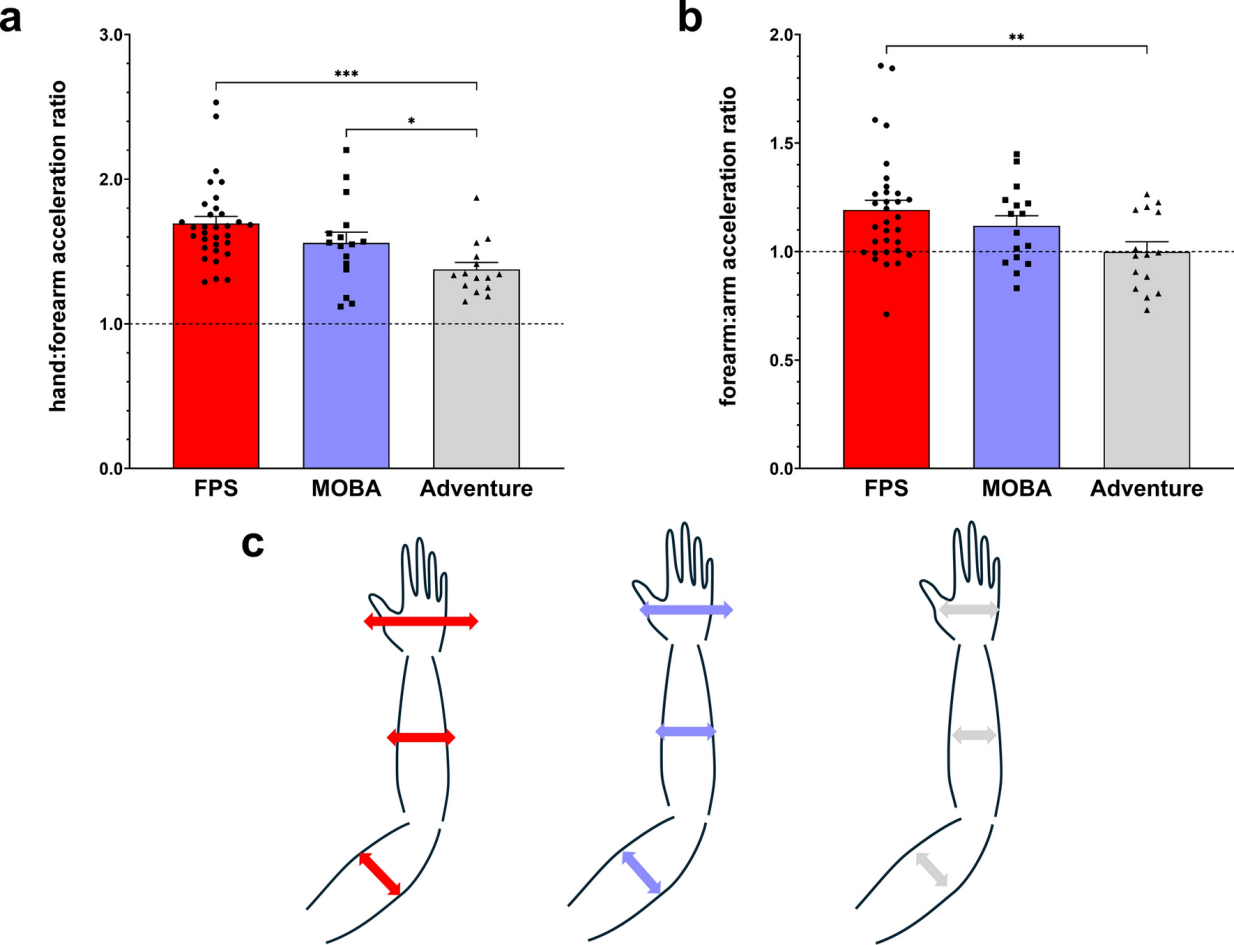
(**a**) Root mean square (RMS) of global hand acceleration relative to forearm ratio (hand:forearm) (mean ± SEM) for *FPS* (red), *MOBA* (blue), and *Adventure* (grey) players. (**b**) RMS of global forearm acceleration relative to arm acceleration ratio (forearm:arm) in function of genre (mean ± SEM) for *FPS* (red), *MOBA* (blue), and *Adventure* (grey) players. (**c**) Illustration of hand acceleration (scaled according to the results) at three different upper limb level (hand, forearm, arm) for *FPS* (red), *MOBA* (blue), and *Adventure* (grey) players. *, ** and *** indicate significant differences at p ≤ 0.05, p ≤ 0.01 and p ≤ 0.001, respectively.

A significant main effect of genre was found when examining differences between hand:forearm RMS acceleration ratios (F_(2,60)_ = 8.058, p = 0.001, η^2^ = 0.212) (Fig. 2a,b). Post hoc analyses revealed that the hand:forearm acceleration ratios among FPS players were greater than those for both MOBA (p ≤ 0.043) and Adventure players (p = 0.001). No significant difference in this ratio was found between MOBA and Adventure players (p = 0.102).

A main effect of genre was also found for the RMS acceleration ratio between the forearm and arm (forearm:arm) (F_(2,60)_ = 4.414, p = 0.016, η^2^ = 0.128) (Fig. 2a,c). Post hoc analyses revealed that the forearm:arm acceleration ratio among FPS players was greater than that for Adventure players (p = 0.004). No significant difference in forearm:arm ratio was found between FPS and MOBA players (p = 0.299) or between MOBA and Adventure players (p = 0.096).

### Hand direction changes (repetitive motion)

**Fig. 3:**
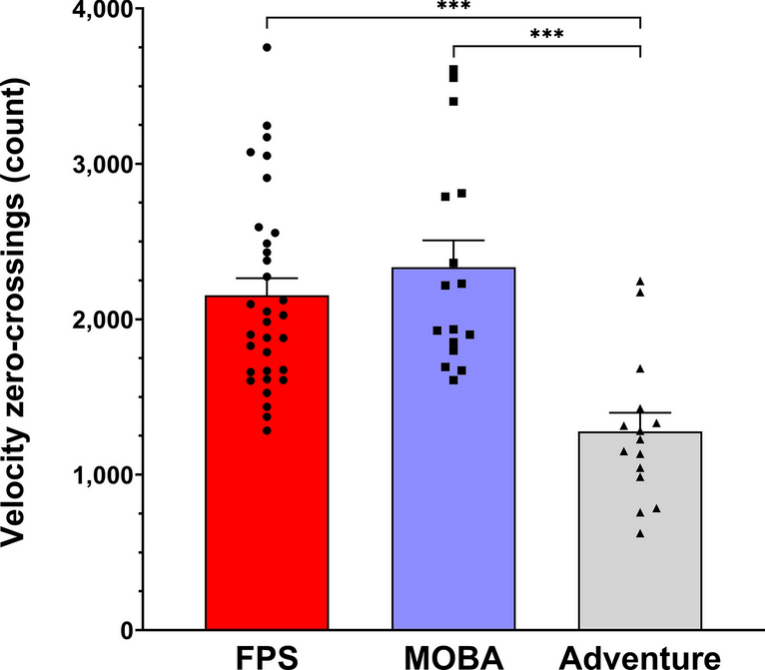
Hand global velocity signal zero-crossings during the 10-min gameplay (mean ± SEM) for *FPS* (red), *MOBA* (blue), and *Adventure* (grey) players. *, ** and *** indicate significant differences at p ≤ 0.05, p ≤ 0.01 and p ≤ 0.001, respectively.

A main effect of game genre was found for the total number of global velocity zero-crossings (F_(2,60)_ = 21.468, p ≤ 0.001, η^2^ = 0.417) (Fig. 3). Post hoc analyses revealed that FPS (p ≤ 0.001) and MOBA players (p ≤ 0.001) had a higher number of velocity zero crossings compared to Adventure players. No significant difference was found between FPS and MOBA players (p = 0.376).

### Hand displacement

#### Cumulative distance travelled

**Fig. 4:**
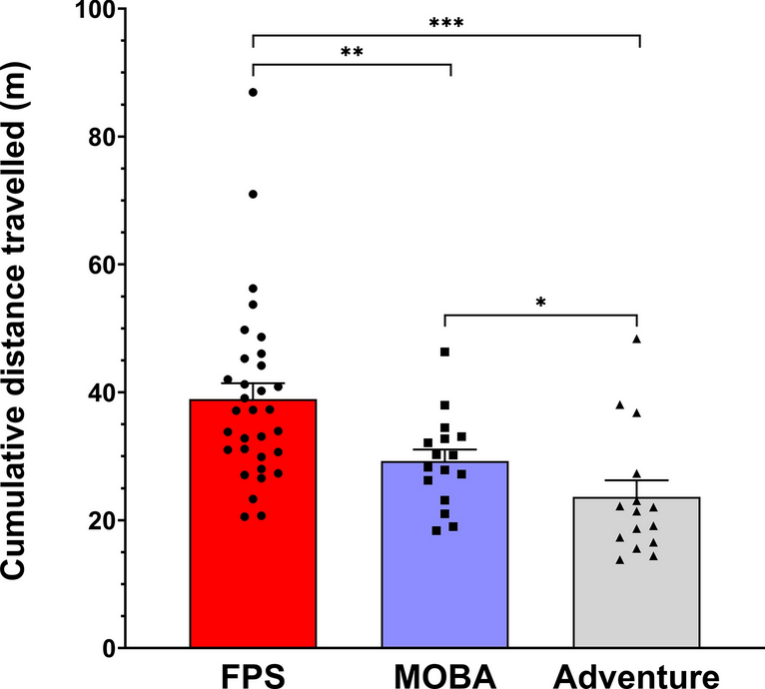
Hand cumulative distance travelled (mean ± SEM) for *FPS* (red), *MOBA* (blue), and *Adventure* (grey) players. *, ** and *** indicate significant differences at p ≤ 0.05, p ≤ 0.01 and p ≤ 0.001, respectively.

A significant main effect of genre was found for cumulative travel distance of the hand (F_(2,60)_ = 13.873, p ≤ 0.001, η^2^ = 0.316) (Fig. 4). Post hoc analyses showed that FPS players moved their hand through a greater distance compared to both MOBA (p = 0.010) and Adventure players (p ≤ 0.001). Additionally, MOBA players moved their hand through a greater distance compared to Adventure players (p = 0.03).

#### Displacement area (size and shape)

**Fig. 5:**
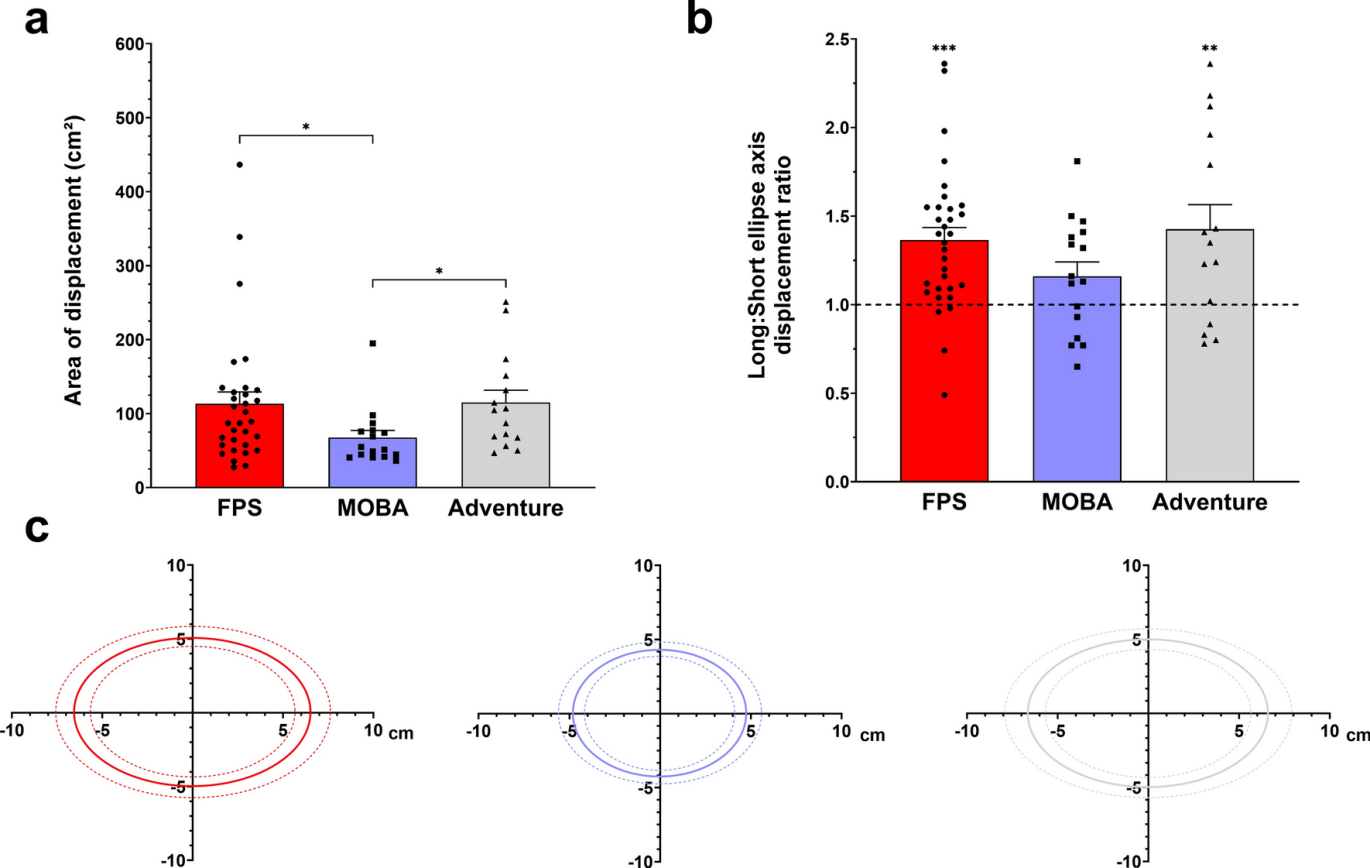
(**a**) Hand displacement area (mean ± SEM) for *FPS* (red), *MOBA* (blue), and *Adventure* (grey) players. (**b**) Long:Short displacement ratio (mean ± SEM) for *FPS* (red), *MOBA* (blue), and *Adventure* (grey) players. **c.** Illustration of the hand displacement area (mean displacement = solid lines; 95%CI = dotted lines) for *FPS* (red), *MOBA* (blue), and *Adventure* (grey) players. *, ** and *** indicate significant differences at p ≤ 0.05, p ≤ 0.01 and p ≤ 0.001, respectively.

When comparing the areas over which gamers moved their hand (Fig. 5a,c), a main effect of game genre was found (F_(2,60)_ = 3.338, p = 0.042, η^2^ = 0.100). Post hoc analyses showed that FPS (p = 0.018) and Adventure (p = 0.04) players moved their hand over a greater area on the mousepad compared to MOBA players. No significant difference was found between FPS and Adventure players (p = 0.838). Upon observing that as an exploratory analysis, we also investigated the ellipse shape (Ratio of ellipse long and short axes (Long:Short)) (Fig. 5b,c). When comparing the Long:Short ratio to 1 (perfect circle) using one-sample t-tests for each game genre group, FPS (t_(31)_ = 5.154, p ≤ 0.001, η^2^ = 0.172), and Adventure (t_(14)_ = 3.073, p = 0.008, η^2^ = 0.135) players’ had ratios that were significantly different from 1, while no significant difference was found among MOBA players (t_(15)_ = 1.977, p = 0.067, η^2^ = 0.067).

## Discussion

This study is the first to demonstrate that kinematic behaviours in esports vary significantly by game genre, highlighting unique biomechanical demands across FPS, MOBA, and Adventure games. Overall, we observed different kinematic patterns among FPS, MOBA, and Adventure players over the same period of gameplay.

As stated by Campbell and colleagues, and more recently by Manci and colleagues, the cognitive skills required to perform in esports may differ according to the game genre^4,12^. Our study highlights for the first time that game genres can be differentiated according to their biomechanical demands. More specifically, players show different levels of ballistic hand movements based on the game genre they played. Specifically, FPS players moved their hand with greater acceleration/deceleration magnitudes compared to players from other genres (23% and 49% more than MOBA and Adventure players, respectively). In turn, MOBA players exhibited 34% greater acceleration/deceleration magnitudes compared to Adventure players. Just as different athletic disciplines demand specific kinematics (sprint, hurdles, jumps, etc.), video game play also require unique kinematics based on the game genre (FPS, MOBA, Adventure, etc.). In most FPS games, such as Counter Strike, enemy encounters typically lasts less than a second, making quick and accurate movements crucial to successfully eliminate opponents. In contrast, Adventure games prioritize the accuracy of decisions and strategy development. While the difference in hand acceleration magnitude between FPS and MOBA players may be partially attributed to differences in DPI choice, it could also be influenced by the perspective with which players experience their game environment (first-person view in FPS games vs. isometric view in MOBA games). In FPS games, players must look where they want to aim, often scanning left and right to avoid ambushes. In contrast, MOBA players have a pre-determined view of the area around their avatar, allowing them to rely less on large changes of direction to gain situational awareness. These findings emphasize the importance of designing custom deliberate training drills and exercises based on the game genre in which players perform. For instance, aim trainers have been shown to be reliable for assessing^32,33^ and improving FPS players’ skills^33^ and could be beneficial for talent identification and training.

When examining the coordinated control of the upper limb, FPS players showed greater disparity in the movements of their upper limb segments to control the mouse compared to MOBA and Adventure players (11 and 22% more hand:forearm RMS acceleration, respectively and 20% more forearm:arm RMS acceleration compared to Adventure players). This may be explained by strategies to increase accuracy and precision of rapid movements. Since the speed-accuracy trade-off theory states that movements that occur at a rapid pace are less accurate^34^, FPS players may increase the degrees of freedom at the wrist joint to leverage proprioceptive sensory feedback associated with mouse and cursor movements. This feedback may facilitate online detection and adjustment of the movement of these segments^35^. This may also explain why FPS players tend to select a lower cursor sensitivity (400-1000 DPI). The proportional error in larger movements (low sensitivity) is smaller than the proportional error of smaller movements (high sensitivity)^35^, which may facilitate accurate movement^36^. A parallel could be drawn between the evolution of motor control throughout infancy and expertise in sports, as young and novice individuals reduce the degrees of freedom at various joints by increasing limb stiffness to decrease movement error rate^36–38^. For instance, Button and coworkers observed that expert basket-ball athletes exhibited more than twice the range of flexion at the wrist during free-throws while being more accurate compared to novice athletes^39^. This information is valuable as it may inform the development of deliberate training exercises^40^ which may encourage a “stiffer” joint strategy among novice players and then dynamically encourage more disparity among upper limb segment movements as expertise develops. However, this topic has not yet been examined and further research is needed to better understand how players acquire specific esports’ motor skills and strategies.

Considering computer-based tasks, previous work has shown that biomechanical strain is largely related to the presence of highly repetitive motion^41–43^. Repetitive submaximal movement can cause tendon microtrauma, tendon sheath irritation, and nerve compression^44^. Our findings show that FPS and MOBA players display 72 and 87% more hand direction changes compared to Adventure players. This may be due to the dynamic and competitive nature of FPS and MOBA games that do not frequently allow players mid-game breaks. These games also demand that players continually move to avoid becoming an easy target for opponents, likely resulting in more actions per minute and more frequent hand-mouse direction changes. In addition, as discussed above regarding upper limb segment acceleration ratios, MOBA players tend to have a more rigid control strategy (ratio closer to 1) of their upper limb segments while exerting many hand direction changes. These kinematic motor patterns may suggest increased co-contraction of upper limb muscles to stabilize the joints^45,46^ and may derive from the tendency of MOBA players to choose higher cursor sensitivities. This stiffer control strategy may cause more frequent joints oscillations^47^ which may lead to more direction changes and ultimately contribute to increasing repetitive strain injury risk. Sousa and colleagues highlighted that training for 2 h without breaks increases impulsive decision-making^48^ which may hinder performance, while Difrancisco-Donoghue and colleagues showed that executive function and processing speed improves after 6 min of walking^49^. As esports players can practice between 5.5 and 12 h per day^44,50–54^, identifying in which genres players are at increased risk of experiencing pain is important for the development and implementation of preventative measures to mitigate pain and injury. For instance, including additional breaks^49,55^ with pain avoidance strategies (stretching^56^, physical activity, and conditioning^57^) and incorporating smaller spaced-out times for deliberate training instead of the traditional focus on long continuous bouts of practice may improve players training experience^33,40,42,44^. The benefit that deliberate and efficient training sessions may not only extend to performance but may also mitigate risk of pain and injury as well among FPS and MOBA players.

Our findings that movement differs among players of different game genres also has implications for gaming equipment. We saw that FPS players moved their hand through a greater distance compared to Adventure players (67% more) and MOBA players (30% more), while MOBA players moved their hand through a greater distance compared to Adventure players (29% more). Interestingly, gaming and esports manufacturers often evaluate the durability and recommended replacement time of their gaming mouse feet based on the maximal mouse travel distance. In fact, the mouse feet used in this experiment (Logitech Pro Wireless PTFE feet) had a durability equivalent of over 250 kilometers^58^. Considering that professional FPS players are reported to train for an average of 50 h a week^24^, and that FPS players in our study moved their hand through a distance of 39 m over only 10-min of gameplay, this would suggest their PTFE mouse feet would significantly degrade in quality and require replacement after approximately 6 months. This information could be used to better inform players and teams as to the lifespan of their gaming equipment and better inform manufacturers regarding the real-world limits of their products.

In addition to the mouse, our findings may have implications for the design of mousepads as well. We observed that Adventure and FPS players’ hand displacements encompassed a 38% larger area of the mousepad compared to MOBA players. Likewise, we have noticed that hand displacement areas were more oblong for Adventure and FPS players compared to MOBA players whose movements were contained within a more circular area. These findings could be used as an index to determine the minimum requirements for mousepad design and sizing for specific game genres. For instance, FPS and Adventure players may benefit from using larger mousepads, while MOBA players could perform effectively on smaller mousepads. Manufacturers could enhance the player experience by designing peripherals (mice and mousepads) that are directly informed by the biomechanical demands of each game genre. For example, peripherals could be tailored according to hand and forearm accelerations and decelerations to improve control for FPS players and support MOBA players with changes in direction.

An important consideration in the explanation of the different movement characteristics displayed by players of different genres is the DPI used. It may be argued that larger, more rapid hand movements displayed by some players may be purely due to their selection of lower cursor sensitivities rather than an inherent demand of the game genre. While DPI undoubtedly does play a role, we however found that MOBA players moved a greater distance in a smaller area than Adventure players despite having no significant difference in DPI choice. This supports the assertion that specific game demands do factor into the unique kinematics among players of different game genres. When considering FPS player kinematics, some of the patterns we observe may be explained by the lower DPI used by FPS gamers. However, FPS games notoriously place high demands on precision and accuracy in short time windows^12,23,24^. As explained above, greater wrist joint degrees of freedom and higher movement magnitudes may afford greater resolution of sensory feedback among FPS players, which would facilitate learning and performance for tasks demanding high accuracy and precision. Overall, while DPI may be a factor, game genre does indeed play an important role for dictating the observed kinematic profiles of esports players.

While this study presents the first demonstration of the biomechanical differences displayed by esports players of different game genres, we do note a few limitations that limit our ability to address certain research questions, and which could be targets of future research. First, the design of the study was such that we focused solely on the kinematics of the right upper limb (mouse side), with no data collected concerning the kinematics of the left upper limb (keyboard control). As many esports skills in genres like FPS and MOBA require bimanual coordination, it would be interesting to understand how game genres differ in their demands to keyboard control as well^59^. Secondly, our study did not investigate how movement kinematics within a game genre differed according to the expertise of each player. Future studies of biomechanical differences among players within single esport genres would provide additional value relating to training and talent identification^23,24^. Finally, we observed esports players during 10 min of gameplay while typical game times can vary across genres. Consequently, future work could look to investigate kinematics over longer gameplay times to capture the effect of elements like peripheral fatigue among players of various game genres.

## Conclusion

This study demonstrated, for the first time, that different game genres place unique physical demands on players that affects their kinematic behaviour. FPS players exerted the most rapid movement and the most distance travelled in a large and oblong mousepad area. Conversely, MOBA players showed a high number of hand direction changes in a smaller area of the mousepad. Finally, Adventure players displayed the lowest magnitude of acceleration, distance travelled, direction changes, but moved within a large and oblong area comparable to the FPS players area of displacement. These findings provide unique insights into the kinematic demands of diverse game genres and lay the groundwork for game genre-specific research in esports biomechanics. Ultimately, understanding the biomechanical factors associated with various game genres will enable the development of tailored training guidelines and support the identification of potential injury risks specific to each esports genre. This, in turn, will inform the design of preventative strategies adapted to players’ needs and guide manufacturers in the design of gaming equipment that may enhance player experience and performance.

## Methods

### Participants

Sixty-three (N = 63) participants who attended Gamescom 2023 in Köln, Germany, were recruited to participate in the study. Participants were included if they had no diagnosed neurocognitive disorders, no previous upper limb injuries in the past year, and had at least 100 h of total game time experience in the video game genre they chose to play in the experiment. All participants were right-handed and used a mouse in their right hand. All participants were informed of how data was intended to be collected and gave their written consent to participate to the study. The experiment was approved by the University of Limerick research ethics board in accordance with the Declaration of Helsinki (2023_06_17_EHS).

### Procedures

#### Demographics and settings

**Fig. 6:**
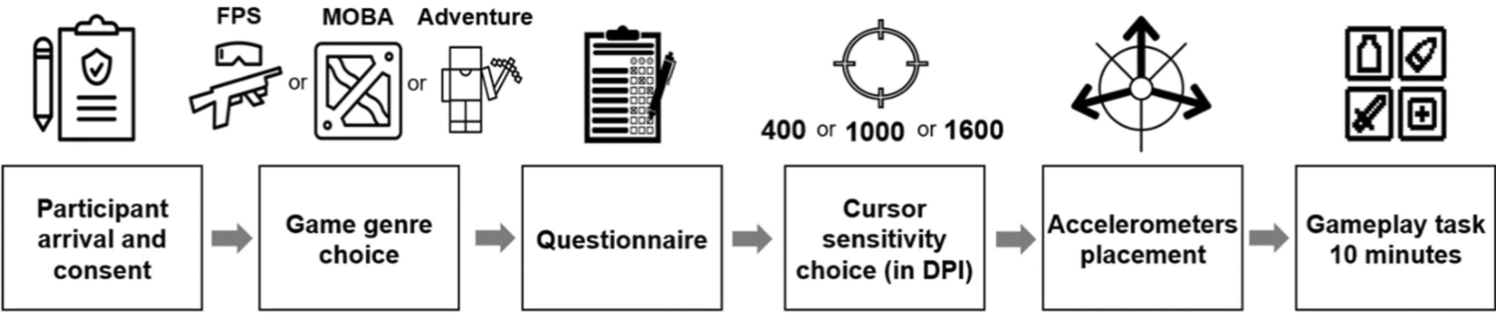
Experimental sequence design. *DPI* Dots Per Inch, *FPS* First-Person Shooter, *MOBA* Multiplayer Online Battle Arena.

Participants chose to play a typical FPS game (Counter-Strike: Global Offensive (CS:GO); FPS group, n = 32), a MOBA game (League of Legends (LoL); MOBA group, n = 16), or an Adventure game (Minecraft; Adventure group, n = 15). They completed a questionnaire that captured information about participant demographics, anthropometrics, and gaming habits. Finally, participants chose one of 3 different DPI (400 DPI, 1000 DPI, and 1600 DPI) and played with the default in-game cursor sensitivity and settings (Fig. 6). Cursor sensitivity refers to the distance the cursor travels over the screen relative to the physical distance that the computer mouse travels over the mousepad. The participants allocated to the FPS, MOBA and Adventure groups were aged 23.7 (± 5.0), 25.6 (± 4.2), and 22.5 (± 5.1) years, respectively. The average number of gameplay hours per week for FPS, MOBA, and Adventure players were 18.3 (± 13.5), 16.9 (± 12.2), and 20.9 (± 8.5), respectively. Participants used standardized equipment consisting of a Logitech Pro Wireless mouse, a Logitech soft mat (45 cm × 40 cm), and a Logitech Pro keyboard. Regarding the PC configuration, a gaming laptop (Lenovo Legion S5-13700H/32/512/4070 16IRH8, NVIDIA GeForce RTX) was connected to a monitor (Samsung C27G75 Odyssey G75/27/2560 × 1440/Curved, 27′′, 144 Hz).

#### Upper limb kinematics: accelerometers

Prior to their 10 min of gameplay, three wireless triaxial accelerometers (Noraxon Inc., Scottsdale, AZ, USA, EMG sensor Model 810) were placed on the dorsal side of participants’ right-hand (between the distal head of the 3rd metacarpal process, the proximal base of the 2nd metacarpal process, and the proximal base of the 4th metacarpal process with the + x-axis aligned with the 3rd metacarpal bone), forearm (half-distance from the olecranon fossa and the imaginary line between the ulnar and radial styloid process with the + x-axis aligned along the forearm and pointing to the wrist), and arm (half-distance between the acromion and the lateral epicondyle with the + x-axis aligned in the continuation of the arm and pointing to the elbow). Accelerometers placed on the forearm and the arm were secured using an elastic strap to minimize motion due to skin artefact. The accelerometers were placed to represent specific segments of the right upper limb (hand, forearm, and arm). These locations were chosen because it represented the midpoint of each limb which is approximately where the centre of mass is situated, and to avoid recording interfering motions between segments (see Supplementary Fig. S1 online). Analog data captured by the accelerometers were wirelessly sent to a receiver (Noraxon 880 Ultium) that sampled the data at 500 Hz via Noraxon myoRESEARCH® (MR 3.21) software.

#### Task completion

After the accelerometers were secured, the computer mouse was aligned to a small target marked on the centre of the mousepad to standardize mouse starting position. Participants who chose to play FPS played CS: GO in Competitive mode in the Dust II environment against AI bots. Participants who chose MOBA played LoL in the Summoner’s Rift virtual environment (5 vs 5, standard mode) against AI bots (intermediate level). Finally, when playing the Adventure game genre, participants played Minecraft in Survival mode set to “Hard” difficulty to explore, craft tools and weapons, build a shelter, and survive. They were free to choose their survival method and explore the world but were instructed to remain active in the game. All participants played their chosen game for a maximum of 10 min and were instructed to play their best during gameplay.

### Data processing

The data pre-processing and processing were performed by a custom Python script (Data Availability).

#### Pre-processing

Pre-processing of accelerometery data was performed separately for each axis (x, y, z) for each accelerometer. Firstly, outlier acceleration data ± 3 standard deviations (SDs) beyond the mean (average data unchanged = 98.9% ± 0.42) were replaced using linear interpolation method^60^. After outlier removal and replacement, accelerometery data were zeroed to remove the component of gravity by subtracting the global 10-min mean acceleration value across the 10-min of gameplay from the value in each frame. Subsequently, as raw accelerometery data were recorded in mG (milli gravitational force), raw data were converted to meters per second squared (m.s^-2^). Then, data were filtered using a 2nd order bandpass Butterworth filter with a 0.5–11 Hz passband^61,62^ (see Supplementary Fig. S2 online). To calculate velocity, the acceleration signals (x, y, and z) were integrated using a cumulative trapezoid method. This step was also performed on the velocity data (2nd integration step) to then provide changes in displacement across the session. After performing these pre-processing steps, we calculated the global acceleration and displacement of the hand, forearm and arm using the respective x, y and z accelerations, and displacements as in Eqs. (1) and (2).

1 $$Acceleratio{n}_{Limb}=\sqrt{{ax}_{{Limb}_{n}}^{2}}+ \sqrt{{ay}_{{Limb}_{n}}^{2}}+\sqrt{{az}_{{Limb}_{n}}^{2}}$$

2 $${Displacement}_{Limb}=\sqrt{{dx}_{{Limb}_{n}}^{2}}+ \sqrt{{dy}_{{Limb}_{n}}^{2}}+\sqrt{{dz}_{{Limb}_{n}}^{2}}$$

where ${ax}_{{Limb}_{n}}^{2}$ represents an acceleration and ${dx}_{{Limb}_{n}}^{2}$ a displacement value along the x-axis at a given time point, ${ay}_{{Limb}_{n}}^{2}$ represents an acceleration and ${dy}_{{Limb}_{n}}^{2}$ a displacement value along the y-axis, and ${az}_{{Limb}_{n}}^{2}$ represents an acceleration and ${dz}_{{Limb}_{n}}^{2}$ a displacement value along the z-axis. $Acceleratio{n}_{Limb}$ (units m.s^-2^) and ${Displacement}_{Limb}$ (m) represent the global acceleration and displacement (irrespective of the axes) of a specified limb across the 10 min of gameplay respectively.

##### Processing

To address each aim, the following variables were calculated from the pre-processed data: global hand RMS acceleration magnitude, hand RMS acceleration relative to forearm and forearm relative to arm ratios, number of hand velocity zero-crossings, hand cumulative distance travelled, and the displacement area of the hand represented by a 95% confidence ellipse.

Firstly, we calculated the RMS of the hand acceleration as in Eq. (3) where ${Acceleration}_{{hand}_{i}}$ represents all acceleration value from Eq. (1), and $n$ represents the total number of data frames. ${Hand}_{rms}$ is expressed in m.s^-2^ and represents the acceleration magnitude across the 10 min of gameplay.

We used the same method to calculate the RMS of both forearm and arm acceleration.

3 $${Hand}_{rms}=\sqrt{\frac{1}{n}\sum_{i=1}^{n}{{(Acceleration}_{{Hand}_{i}})}^{2}}$$

Subsequently, we calculated the ratio of hand RMS acceleration relative to forearm RMS acceleration (hand:forearm) and the ratio of forearm relative to arm RMS acceleration (forearm:arm) using the global RMS acceleration from Eq. (3) for each limb as in Eqs. (4) and (5):

4 $$hand:forearm= \frac{{Hand}_{rms}}{{Forearm}_{rms}}$$

5 $$forearm:arm= \frac{{Forearm}_{rms}}{{Arm}_{rms}}$$

To determine the number of changes in hand direction over the course of gameplay, the number of zero-crossings in x–y-z axes hand velocity signal were summed across the 10 min.

Next, we calculated the cumulative distance the hand travelled (CD) by adding all absolute changes in displacement (2) across subsequent frames throughout the 10 min of gameplay as in Eq. (6).

6 $$CD = {\sum }_{i=0}^{n}\left|{{Displacement}_{Hand}}_{i+1}\right|- \left|{{Displacement}_{Hand}}_{i}\right|$$

where ${Displacement}_{Hand}$ represents the displacement value at the *ith* frame.

Finally, we plotted the x and y displacement coordinates at each frame and determined the area of the 95% confidence ellipse of these displacements as in Eq. (7).

7 $$A=\pi ab$$

where $A$ represents the area of the ellipse, $a$ represents the length of the ellipse long axis and $b$ represents the length of ellipse short axis.

### Statistical analysis

Analyses were conducted using SPSS v29 software (SPSS Inc., Chicago, Ill., USA). Means and SDs are reported for participants’ age and gameplay time (in hours per week). To determine whether players of different game genres chose different cursor sensitivities, we performed a contingency analysis (Chi-square analysis) with Sidak’s correction on participants DPI choice. Data normality and homogeneity of variance were tested using Shapiro–Wilk test and Levene’s test respectively. Because all data were not normally distributed, we performed a log transformation supplemented by a simple sampling bootstrapping method (number of samples = 1000; confidence interval level = 95%) and retested data distribution after log transformation^63^. Once log transformed, data followed a normal distribution (Shapiro–Wilk test) and met the assumption of homogeneity of variance (Levene’s test). One-way (Game Genre) analyses of variance (ANOVAs) were performed to evaluate the influence of game genre on multiple dependent variables. Pairwise comparisons were performed using Fisher’s Least Significant Difference (LSD) to explore specific group differences^64,65^. Means and standard error (SEM) are reported for all data. Significance was determined where p ≤ 0.05.

## Supplementary Information

Supplementary Information.

## Data Availability

The datasets generated using the methods described and which undertook the described analysis in the current study are openly available in osf.io and can be found at https://doi.org/10.17605/OSF.IO/AHXE4. The custom Python script can be found following this link: https://gist.github.com/Antoine-DupuyUL/79ae8aa2f4153757316c712e0d46ba8a.
