# Supplementary material for: Differentiating right upper limb movements of esports players who play different game genres
Source: Sci Rep. 2025 Feb 22;15:6498. doi: 10.1038/s41598-025-90949-6 (PMC11846987; doi:10.1038/s41598-025-90949-6)
Supplement: Supplementary file 1 — Supplementary Information. [file 41598_2025_90949_MOESM1_ESM.docx]

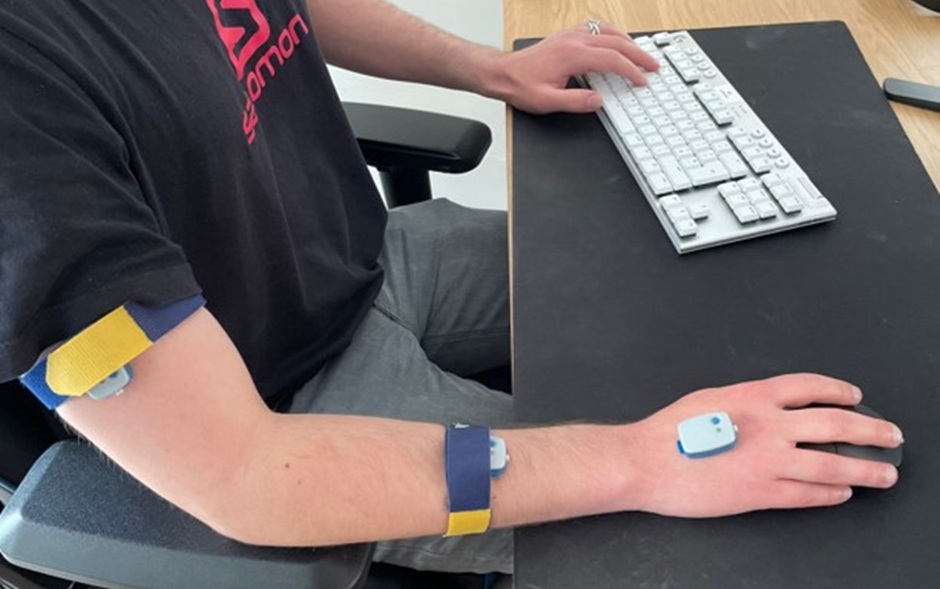


**Supplementary Figure S1:** Depiction of accelerometer placements on right upper limb segments.


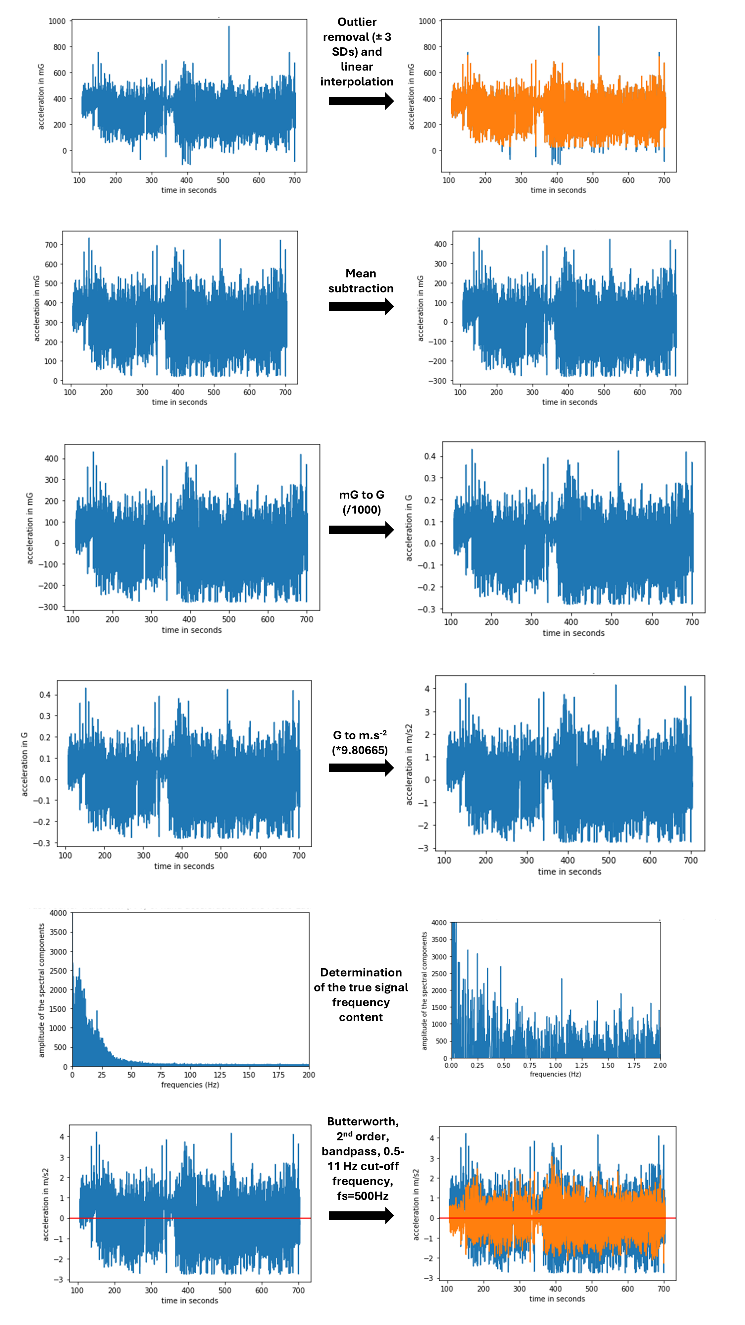


**Supplementary Figure S2:** Python processing steps implemented on raw accelerometer data.
